# DNA METHYLATION AT THE CROSSROADS OF PHYSIOLOGICAL RESPONSE TO STRESS AND PATHOLOGICAL BIOLOGY

**DOI:** 10.1093/geroni/igz038.2694

**Published:** 2019-11-08

**Authors:** Shabnam Salimi

**Affiliations:** University of Maryland Baltimore School of Medicine, Baltimore, Maryland, United States

## Abstract

It is likely that the dimension and the scope of stress perception are different in different body systems conditioning their specific rates of aging. Moreover, in addition to between-individual variabilities in body response to stress, there is inter-organs variability in adaptations resulting in pleiotropy of phenotypes evolved with aging. Epigenetic measures may be considered possible readouts of the extent and proxy of the adaptive response to intrinsic and/or extrinsic stress in body organs. It is thus important to disentangle the epigenetic basis of stress, adaptation, and pathology. In this symposium we discuss a combination of the specific DNA methylation patterns relate to the diseases as an intrinsic stressor and adaptation responses imposed to bodily systems, and also accelerated body organ morbidities. We explain the static and dynamic DNA methylation differentiation over time as the “epigenetic code” of accelerated pathology across various age using longitudinal data.

